# Programmed Cell Death Protein 1 Inhibitors and MET Targeted Therapies in NSCLC With *MET* Exon 14 Skipping Mutations: Efficacy and Toxicity as Sequential Therapies

**DOI:** 10.1016/j.jtocrr.2023.100562

**Published:** 2023-08-19

**Authors:** Sally C.M. Lau, Kirstin Perdrizet, Andrea S. Fung, Danilo Giffoni M.M. Mata, Jessica Weiss, Nick Holzapfel, Geoffrey Liu, Penelope A. Bradbury, Frances A. Shepherd, Adrian G. Sacher, Harriet Feilotter, Brandon Sheffield, David Hwang, Ming Sound Tsao, Susanna Cheng, Parneet Cheema, Natasha B. Leighl

**Affiliations:** a Department of Medical Oncology, Princess Margaret Cancer Centre, University Health Network, University of Toronto, Toronto, Ontario, Canada; b Department of Medical Oncology, Laura and Isaac Perlmutter Cancer Center, New York University (NYU) Langone Health, NYU Grossman School of Medicine, New York, New York; c William Osler Health System, Brampton Civic Hospital, Brampton Ontario, Canada; d Department of Medical Oncology, Cancer Centre of Southeastern Ontario, Kingston Health Sciences Centre, Queen’s University, Kingston, Ontario, Canada; e Department of Medical Oncology, Odette Cancer Centre, Sunnybrook Health Sciences Centre, University of Toronto, Toronto, Ontario, Canada; f Department of Biostatistics, Princess Margaret Cancer Centre, University Health Network, University of Toronto, Toronto, Ontario, Canada; g Department of Immunology, University of Toronto, Toronto, Ontario, Canada; h Department of Pathology, Cancer Centre of Southeastern Ontario, Kingston Health Sciences Centre, Queen’s University, Kingston, Ontario, Canada; i Department of Pathology, Sunnybrook Health Sciences Centre, University of Toronto, Toronto, Ontario, Canada; j Department of Pathology, Princess Margaret Cancer Centre, University Health Network, University of Toronto, Toronto, Ontario, Canada

**Keywords:** NSCLC, MET inhibitors, MET exon 14 skipping, PD1 inhibitors

## Abstract

**Introduction:**

NSCLC with *MET* exon 14 skipping mutation (*MET*ex14) is associated with poor outcomes. Integration of novel targeted therapies is challenging because of barriers in testing and drug access. We, therefore, sought to characterize the treatment patterns, outcomes, and emerging issues of treatment sequencing in patients with *MET*ex14-mutant NSCLC.

**Methods:**

We reviewed all NSCLC cases with *MET*ex14 alterations between 2014 and 2020 across four Canadian cancer centers. Demographics, disease characteristics, systemic therapy, overall response rates (ORRs), survival, and toxicity were summarized.

**Results:**

Among 64 patients with *MET*ex14-mutant NSCLC, the median overall survival was 23.1 months: 127.0 months in stage 1, 27.3 months in resected stage 2 and 3, and 16.6 months in unresectable stage 3 or 4 disease, respectively. In patients with advanced disease, 22% were too unwell for systemic treatment. MET tyrosine kinase inhibitors (TKIs) were administered to 28 patients with an ORR of 33%, median progression-free survival of 2.7 months, and 3.8 months for selective TKIs. Programmed cell death protein-1 (PD-1) inhibitors were given to 25 patients—the ORR was 44% and progression-free survival was 10.6 months. No responses were seen with subsequent MET TKIs after initial TKI treatment. Grade 3 or higher toxicities occurred in 64% of patients who received MET TKI after PD-1 inhibitors versus 8% in those who did not receive PD-1 inhibitors.

**Conclusions:**

Many patients with advanced *MET*ex14 NSCLC were too unwell to receive treatment. PD-1 inhibitors seem effective as an initial treatment, although greater toxicity was seen with subsequent MET TKIs. Thus, timely testing for *MET*ex14 skipping and initial therapy are imperative to improving patient survival.

## Introduction

The *MET* exon 14 (*MET*ex14) skipping mutation is a primary oncogenic driver in NSCLC, occurring in approximately 3% to 4% of all cases and up to 32% of pulmonary sarcomatoid tumors.^1^ Unlike other common oncogenic driver subgroups with lung cancer, patients with *MET*ex14 skipping mutations tend to be older, have a smoking history, and are typically diagnosed at a later disease stage.^2^^,^^3^ The presence of *MET*ex14 skipping mutations is associated with poor treatment outcomes.^3^, ^4^, ^5^ Several novel therapies have been recently introduced in this space.^6^, ^7^, ^8^, ^9^ However, the integration of these therapies into clinical practice has been met with challenges, including timely identification of patients with *MET*ex14 skipping mutant NSCLC. *MET*ex14 alterations can arise by means of several genomic alterations such as point mutations, insertions, deletions, and fusions that lead to alternate splicing of the ubiquitin ligase binding site encoded by *MET*ex14.^10^ Without ubiquitination, the MET receptor is not degraded, leading to sustained oncogenic signaling. Clinically, the diverse genomic alterations that lead to the same phenotype create unique challenges in identifying patients with *MET*ex14 skipping mutations, because some may be missed by DNA hot spot panels.^3^^,^^10^

Current first-line treatment for patients with advanced NSCLC includes programmed cell death protein-1 (PD-1) immune checkpoint inhibitors (ICIs) either as monotherapy or in combination with chemotherapy. The pivotal trials establishing PD-1 inhibitors as standard therapy included patients with known *MET*ex14 skipping mutations and those without next-generation sequencing (NGS) who were presumed to have wild-type tumors.^11^, ^12^, ^13^, ^14^, ^15^ Given the low prevalence of *MET*ex14 skipping mutations in NSCLC, treatment responses have not been separately analyzed in clinical studies. In retrospective series, ICI therapy seems to be quite effective among patients with NSCLC with sarcomatoid histology, enriched for *MET*ex14 skipping mutations.^16^^,^^17^ However, other studies of patients with *MET*ex14 skipping mutations among all lung histologies reported conflicting findings of poor results with ICI therapy.^18^^,^^19^

Nonselective multikinase inhibitors crizotinib and cabozantinib have been used as targeted therapies for patients with *MET*ex14 skipping NSCLC with response rates of approximately 30% at best.^9^ Recently, two selective MET tyrosine kinase inhibitors (TKIs), capmatinib, and tepotinib, exhibited efficacy with response rates ranging from 44% to 68% in treatment-naive patients and 48% in previously treated patients.^7^^,^^8^ These have received accelerated approval from the U.S. Food and Drug Administration in both the treatment-naive and previously treated patients. Tolerability also seems improved with selective TKIs.^7^^,^^8^

Selective MET TKIs can be challenging to access in many jurisdictions, and ways to sequence TKIs with existing therapies such as ICI and chemotherapy remain uncertain. There is limited to no prospective data comparing PD-1 inhibitors and selective MET TKIs in this patient population. Whereas MET TKIs exhibit good response rates and tolerability, the data on durability and long-term benefits are immature.^7^^,^^8^ ICI therapy has the potential for long-term survivors in NSCLC, but it is unclear whether this can be extrapolated to patients with tumor *MET*ex14 skipping mutations.^11^, ^12^, ^13^, ^14^, ^15^ These challenges are compounded by the lack of uniform testing for *MET*ex14 skipping mutations, and restricted access to effective targeted therapies even when mutation status is known. We, therefore, sought to characterize the treatment patterns and outcomes of patients with *MET*ex14 skipping mutant lung cancer across four cancer centers in Canada and to explore the impact of treatment sequencing.

## Materials and Methods

The conduct of this study was approved by the institutional review boards of all participating institutions. Individual patient informed consent was waived by the approving IRB institutional review boards.

### Study Population

All NSCLC cases (any stage) with a *MET*ex14 skipping mutation diagnosed between 2014 and 2020 at four cancer centers in Ontario, Canada (Princess Margaret Cancer Centre, Cancer Centre of Southeastern Ontario, Brampton Civic Hospital, and Odette Cancer Centre) were included in this review. Patients were included only when *MET*ex14 skipping was the primary oncogenic driver. Other *MET* gene alterations such as amplification and MET overexpression, unless co-occurring with a *MET*ex14 skipping mutation, were also excluded.

### Genomic Testing

*MET*ex14 skipping mutations were identified through several methods including NGS of tissue using DNA- and RNA-based assays (TruSight Tumor 26 assay, Ilumina, San Diego CA; Oncomine Comprehensive Assay v3, Thermo Fischer, Waltham, MA; or FoundationOne CDx, Foundation Medicine, Cambridge, MA), or plasma-based NGS with commercially available assays (Guardant360 CDx, Redwood City, CA and FoundationOne LiquidCDx, Foundation Medicine, Cambridge, MA). Programmed death-ligand 1 (PD-L1) assessment was performed using the 22C3 pharmDx assay (Agilent Technologies, Dako, Carpinteria, CA) at all centers.

### Study End points, Data Abstraction

Data on patient characteristics including age, sex, Eastern Cooperative Oncology Group (ECOG) performance status, ethnicity, and smoking history were abstracted, and disease stage, treatment, overall response rates (ORRs) to therapy, disease-free survival (DFS) in early-stage patients, progression-free survival (PFS) in late-stage patients, and overall survival (OS). The data cutoff date for survival analysis was October 2021. All computed tomography imaging was reviewed according to Response Evaluation Criteria in Solid Tumors version 1.1 by the study investigators to determine the ORR. Treatment toxicities were also extracted and graded according to Common Terminology Criteria for Adverse Events version 5.0.

### Statistical Analysis

Patient characteristics and treatment patterns were summarized. DFS was calculated from the time of definitive local therapy. Among patients with advanced disease or recurrent disease, PFS was calculated from the start of each line of systemic therapy until disease progression. The OS in patients with early-stage disease was calculated from the date of diagnosis. For patients with late-stage disease or recurrence, OS was calculated from the date of advanced diagnosis or recurrence until death. All survival analyses were performed using the Kaplan-Meier method and tested for differences using the log-rank test. Multivariable Cox regression was performed to adjust for confounding factors such as line of treatment. All statistical analyses were performed using R version 4.0.2 (R Project for Statistical Computing; available at https://www.R-project.org).

## Results

**Figure 1:**
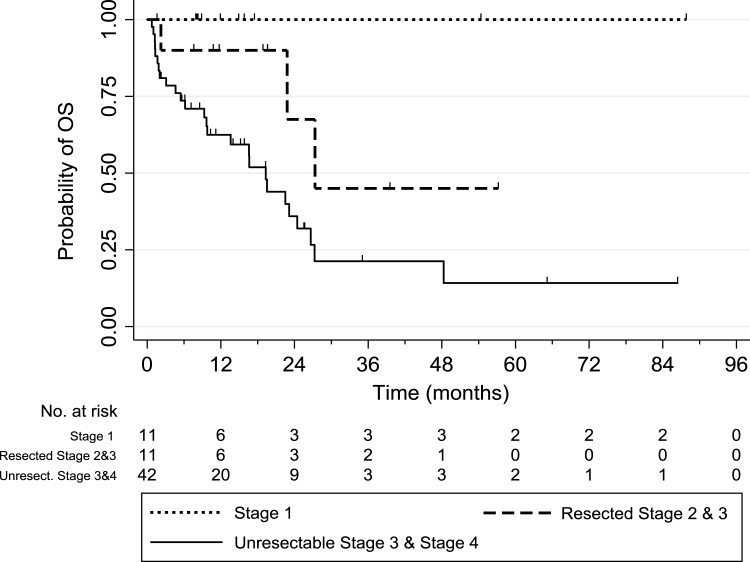
The OS of all included patients with NSCLC having *MET* exon 14 skipping mutations was 23.1 months (95% CI: 16.6–27.3 mo), significantly different by stage at diagnosis: the median OS among those with resected stage 1 disease was 127.0 months; resected stage 2 and 3 disease was 27.3 months; and stage 4 disease was 16.6 months. CI, confidence interval; OS, overall survival.

We identified 64 patients with NSCLC with a *MET*ex14 skipping mutation. The median age of the cohort was 73 years (49–92), 36% were men, 53% were former or current smokers, 80% had adenocarcinoma, and 66% presented with advanced stage (Table 1). PD-L1 expression was known in 63 patients; most (60%) had PD-L1 tumor proportion score greater than or equal to 50%. The specific sites of *MET* alterations were reported in 52 patients: 67% donor splice site mutations, 17% acceptor site mutations, 12% *MET* fusions, and 4% mutations that were not classified, such as deletion of the whole exon. The OS of all patients from the time of disease diagnosis was 23.1 months (95% confidence interval [CI]: 19.3–not reached [NR]) and is summarized by stage in Figure 1.

**Table 1:** Clinical Characteristics of All Included Patients With NSCLC Having *MET* Exon 14 Skipping Mutations

Patient Characteristics	n (%); N = 64
Median age, y	73 (range: 49–92)
Sex	
Female	41 (64)
Male	23 (36)
Ethnicity	
White	32 (50)
Asian	13 (20)
Other or unknown	19 (30)
Smoking	
Nonsmoker	30 (47)
Smoker	34 (53)
Histologic subtype	
Adenocarcinoma	51 (80)
Squamous	2 (3)
Pleomorphic or sarcomatoid carcinoma	7 (11)
Other	4 (6)
Stage at diagnosis	
1	11 (17)
2	3 (5)
3	11 (17)
4	39 (61)
ECOG PS at diagnosis	
0	12 (20)
1	34 (58)
≥2	13 (22)
PD-L1 expression	
≥50%	38 (60)
1%–49%	15 (24)
<1%	10 (16)
*MET* exon 14 aberration	
Acceptor splice site	9 (14)
Donor splice site	35 (55)
Fusion	6 (9)
Other	2 (3)

ECOG PS, Eastern Cooperative Oncology Group performance status; PD-L1, programmed death-ligand 1.

### Treatment and Outcomes in Early-Stage Disease

There were 20 patients who were initially diagnosed with early-stage disease, and treated with stereotactic body radiotherapy or surgical resection and adjuvant platinum-based chemotherapy as indicated per standard of care. Adjuvant ICI was not yet available for this historical cohort of patients. At the time of analysis, 43% of patients had recurred. The median DFS was 29.2 months (95% CI: 11.3–NR), and was 36.1, 12.2, and 7.8 months for patients with stage 1, 2, and 3 diseases, respectively.

### Treatment and Outcomes in Advanced Disease

**Figure 2:**
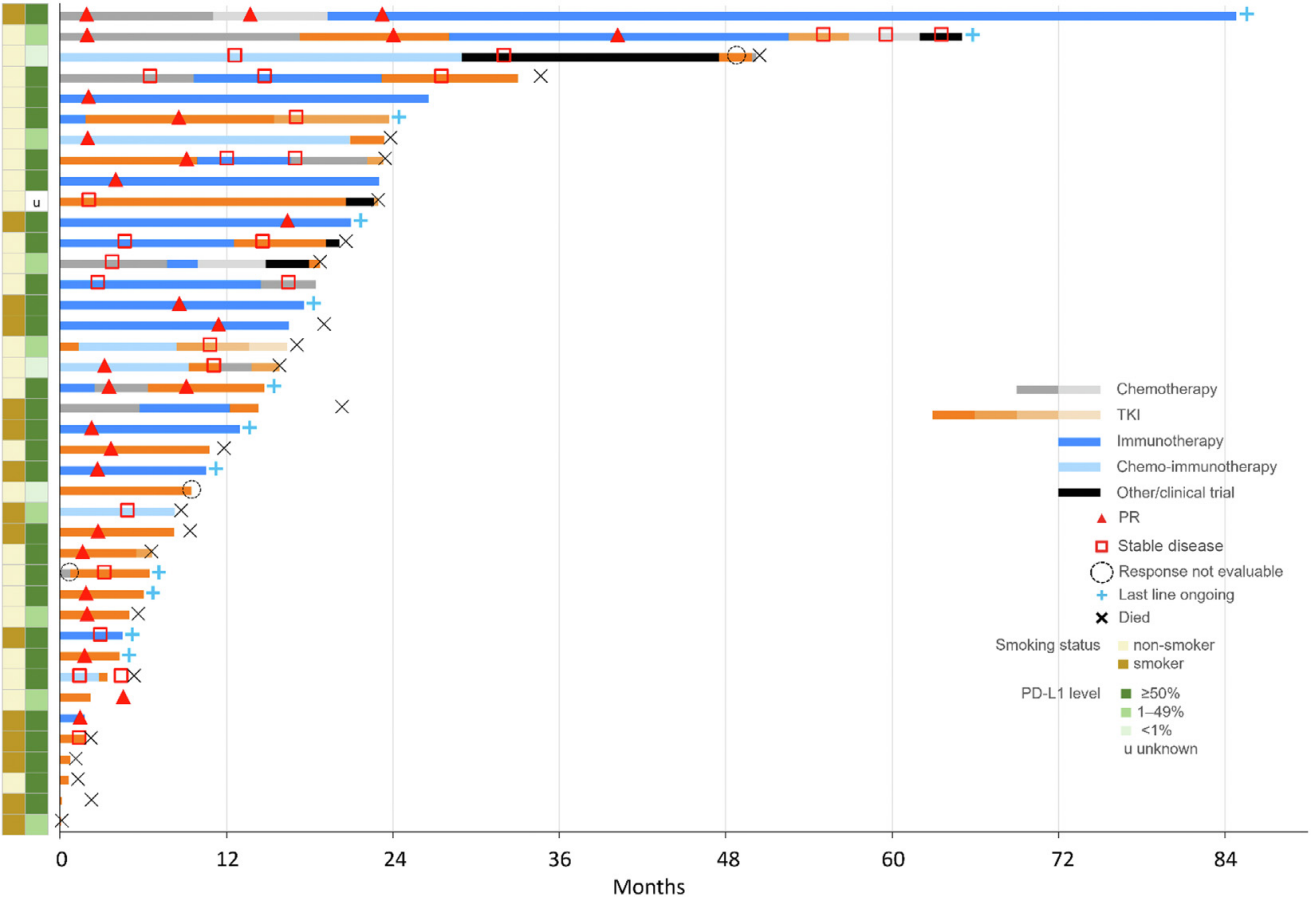
Trajectory of all patients who received any systemic therapy for recurrent or metastatic NSCLC with *MET* exon 14 skipping mutations including the treatment type in sequence, response, and relevant characteristics of smoking history and PD-L1 expression. PD-L1, programmed death-ligand 1; PR, partial response; TKI, tyrosine kinase inhibitors.

A total of 51 patients had de novo metastatic or recurrent disease and were evaluated for systemic therapy. Of these, 22% could not receive any systemic therapy because of poor performance status. The presence of a *MET*ex14 mutation was known in three of these patients, pending in three patients, and retrospectively tested in five patients. Untreated patients had a median OS of 2.0 months (95% CI: 1.2–NR) from the time of metastatic disease diagnosis. Among the 40 treated patients, the median number of systemic therapies received was 1 (range 0–6) and 19 patients received more than one line of therapy. The choice of first-line treatments varied: 40% received crizotinib, 45% received a PD-1 inhibitor alone or in combination, and 15% received chemotherapy alone. In a subset in which NGS results were available at the time of decision-making, 67% of clinicians recommended upfront treatment with a TKI. At progression, 84% of clinicians (16 of 19) chose to switch therapy classes if patients were still fit for systemic therapy. The trajectory of patients with advanced disease and their treatment patterns is summarized in Figure 2. The median OS among the treated patients was 22.5 months (95% CI: 16.6–NR).

### Treatment Response to TKIs

A total of 38 MET TKI treatments were given to 28 patients, including nine patients who received multiple lines of targeted treatments. The characteristics of patients who received TKI treatments are summarized in Supplementary Table 1. Notably, a high proportion of patients (21%) had an ECOG performance status of 2 or higher at diagnosis. Most received a TKI as first-line therapy (n = 16 of 28) and others received TKI as subsequent therapy (n = 12 of 28). The ORR for all TKI treatments combined was 33% (95% CI: 18–49), enriched to 39% (95% CI: 22–59) after excluding those previously treated with another TKI. Selective MET TKIs, capmatinib and tepotinib, were administered to nine patients, all in second-line or beyond settings. The ORR to selective MET TKIs was 33% (95% CI: 9–69) with all responses occurring in patients who had not received previous TKI treatment. The median PFS from the start of MET TKI therapy, including multikinase TKIs, was 2.7 months (95% CI: 2.0–5.2). The median PFS was 3.7 months (95% CI: 1.2–7.6) in patients who received crizotinib first-line, and 3.8 (95% CI: 0.3–4.4) in patients who received newer-generation selective MET TKIs.

### Treatment Response to Immunotherapy

There were 25 patients who received PD-1 inhibitors, alone (76%) or in combination with chemotherapy (24%), and were given as first-line therapy in 72% of patients. The clinical characteristics of ICI-treated patients are summarized in Supplementary Table 2. The ORR was 44% (95% CI: 25–65) in all patients: 54% (95% CI: 26–80) in those receiving single-agent PD-1 inhibitors and 40% (95% CI: 7–83) in those receiving combination immunotherapy. Response rates on the basis of histologic subtypes were similar for adenocarcinoma (44%), sarcomatoid (50%), and other types (33%). The median PFS was 10.6 months (95% CI: 6.3–19.1) in patients receiving immunotherapy with 40% of patients achieving a durable response over 12 months. The clinical and treatment characteristics of long-term responders are summarized in Table 2.

**Table 2:** Characteristics of Patients With a Durable Response to Immunotherapy of Over 12 Months

Patient	Histology	Smoking History	PD-L1, %	Treatment	Tx line	Response	DOR (mo)
PMH4	Pleomorphic or Sarcomatoid	Smoker	≥50	Nivolumab	3	PR	66.7
PMH3	Pleomorphic or Sarcomatoid	Nonsmoker	≥50	Pembrolizumab	1	PR	28.8
PMH17	Adenocarcinoma	Nonsmoker	≥50	Pembrolizumab	1	PR	26.0
WO4	Adenocarcinoma	Nonsmoker	<1	Atezolizumab and chemotherapy	1	Stable disease	24.9
PMH22	Adenocarcinoma	Nonsmoker	1–49	Pembrolizumab	3	PR	19.1
KGH2	NSCLC NOS	Smoker	≥50	Pembrolizumab	1	PR	19.0
PMH6	Adenocarcinoma	Smoker	≥50	Pembrolizumab	1	PR	18.0
PMH13	Adenocarcinoma	Nonsmoker	1–49	Pembrolizumab and chemotherapy	1	PR	17.2
KGH10	Adenocarcinoma	Smoker	≥50	Pembrolizumab	1	Stable disease	15.7
KGH3	Adenocarcinoma	Smoker	≥50	Pembrolizumab	1	PR	13.2

DOR, duration of response; NOS; not otherwise specified; PD-L1, programmed death-ligand 1; PR, partial response; Tx, treatment.

### Treatment Sequencing

**Figure 3:**
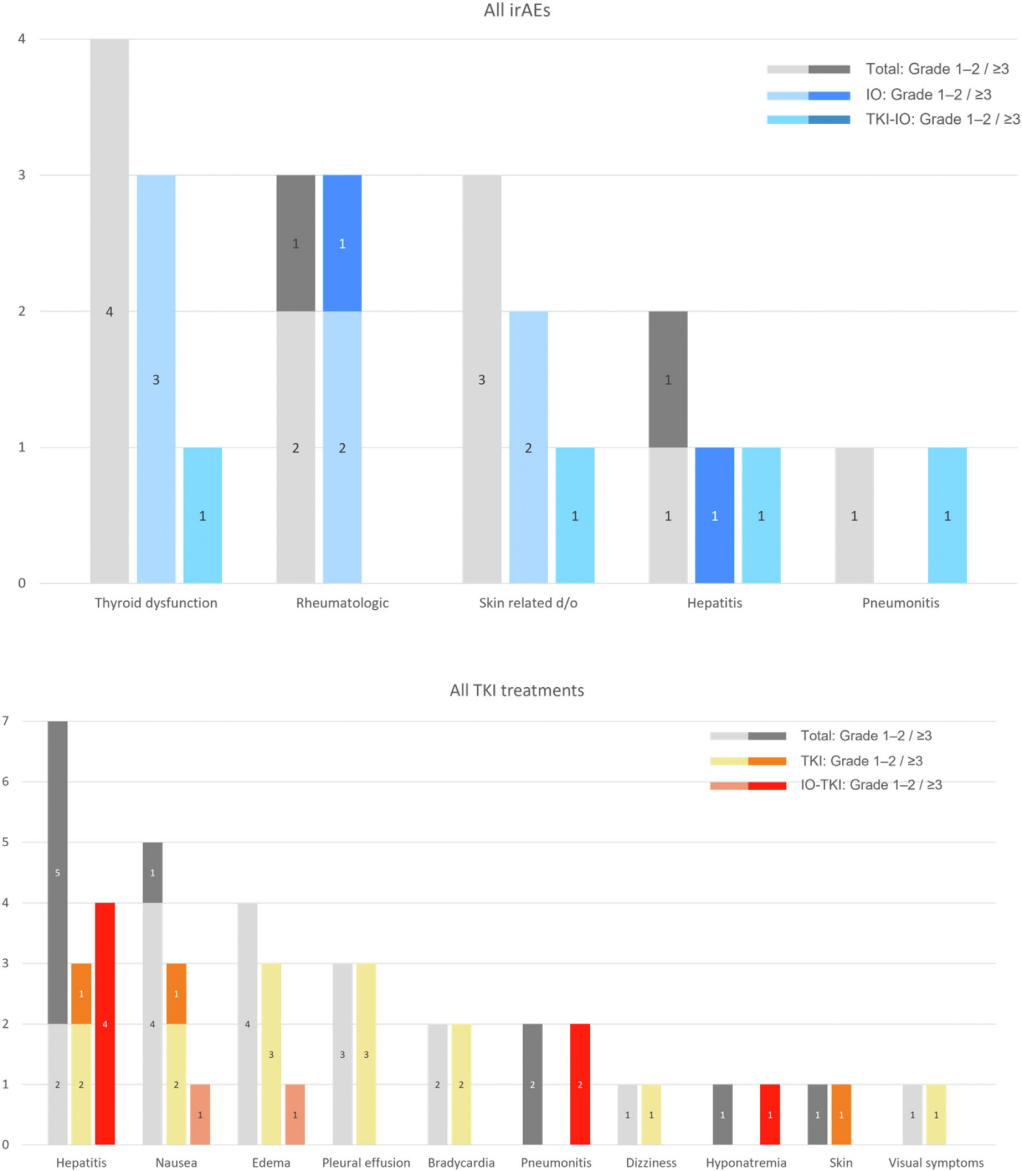
Adverse events and grade of toxicity experienced by patients receiving (*A*) ICIs and (*B*) MET TKIs, stratified by treatment sequence. d/o, disorders; ICI, immune checkpoint inhibitor; IO, immunotherapy; TKI, tyrosine kinase inhibitor.

Exploratory analyses were performed to investigate the effects of treatment sequencing. Among patients who received PD-1 inhibitors as initial therapy, 78% of patients received further treatment at the time of progression, whereas 41% who received a MET TKI as initial therapy received subsequent therapy. The time to second progression was longer in patients who received ICI as part of initial therapy: (17.4 versus 6.1 mo). In a limited subset in which *MET*ex14 skipping mutation was known at the time of diagnosis and ICI was still used as first-line therapy, a similar benefit in the time to second progression was seen (21.9 versus 5.2 mo) (Fig. 3*B*).

There were seven patients who received multiple TKIs, but no partial responses were seen with the second TKI. However, three patients had stable disease, including two patients who received capmatinib after crizotinib.

### Adverse Events

The incidence and treatment-related toxicities are summarized in Figure 3. Treatment was generally well tolerated in patients who received PD-1 inhibitors. The incidence of any immune-related adverse events (AEs) was 44% with ICI therapy (Fig. 3*A*). Grade 3 and higher AEs occurred in 8% of patients and included one case each of hepatitis and dermatomyositis. Toxicity with MET TKIs was common, seen in 64% of all TKI treatment courses, with 27% of patients having grade 3 or higher AEs (Fig. 3*B*).

TKI-related toxicity occurred more typically in patients who received a TKI immediately after an ICI (64% grade ≥3 AE) compared with patients who received a TKI first-line or after chemotherapy or another TKI (8% grade ≥3 AE). The median time from the last ICI to the first TKI was 35 days (range: 16–181 d). The AE details of nine patients who received TKIs as the next line of treatment after ICIs are summarized in Table 3. Permanent discontinuation of TKI was necessary in 33% of patients who received sequential treatment with a TKI after immunotherapy. There were no treatment-related deaths.

**Table 3:** Adverse Events in Patients Who Received a TKI Immediately After ICI

Patient	Drugs	Time to TKI (d)	Adverse Event	Management
PMH20	Pembro and chemo → capmatinib	16	None	
PMH10	Pembrolizumab → campatinib	24	Gr 3 Hepatitis	Dose interruption or reduction
PMH5	Pembrolizumab → crizotinib	30	Gr 3 Hepatitis Gr 4 Hyponatremia	Permanent discontinuation
WO3	Durvalumab → crizotinib	32	None	
WO4	Durvalumab → crizotinib	35	Gr 3 Pneumonitis	Permanent discontinuation
PMH12	Pembro and chemo → crizotinib	82	Gr 4 Pneumonitis	Permanent discontinuation
WO1	Pembrolizumab → crizotinib	91	Gr 3 Hepatitis	Dose interruption or reduction
PMH13	Pembro and chemo → crizotinib	131	None	
PMH8	Pembrolizumab → cabozantinib	181	Gr 3 Hepatitis	Dose interruption or reduction

Chemo, chemotherapy; Gr, grade; ICI, immune checkpoint inhibitor; Pembro, pembrolizumab; TKI, tyrosine kinase inhibitor.

## Discussion

Our study reported on contemporary treatment patterns and outcomes in a large multicenter cohort of patients with *MET*ex14 skipping mutations and exhibited benefits from systemic therapies with survival longer than historically reported. In particular, PD-1 inhibitors were effective as empirical first-line therapy when NGS cannot be done. Treatment outcomes with multikinase inhibitors were worse than expected and newer-generation selective MET TKIs are preferred. Our study was also the first to explore emerging issues of treatment sequencing. Sequential TKIs did not result in additional responses and a switch in the class of therapy (e.g., to chemoimmunotherapy) should be considered at the time of progression. Surprisingly, patients who received PD-1 inhibitors as the first therapy seemed to have longer survival, although we are unable to control for selection bias in this series. High-grade toxicities were frequently encountered when a TKI was given after a PD-1 inhibitor. Although intriguing, optimal treatment sequencing strategies warrant investigation in future studies.

NSCLC with *MET*ex14 skipping mutations is often thought to behave aggressively and several of our findings support this. Patients with resected stage 2 and 3 disease had a short DFS of 11 to 14 months with standard adjuvant chemotherapy. In comparison, wild-type stage 2 or 3 patients in the standard therapy arm of the IMpower010 study had a median DFS of 35 months.^20^ Unfortunately, we could not assess the effectiveness of ICIs in this historical cohort as atezolizumab only gained recent approval in this setting. We also found that a considerable portion (22%) of patients with recurrent or metastatic disease could not receive any systemic therapy because of rapid disease progression or poor performance status. In the real-world setting, treatment outcomes may be much worse than reported in clinical trials in which patients with an ECOG performance status of 2 or higher are often excluded. It is imperative that effective systemic therapy is started as soon as possible. For patients who are able to receive systemic therapy, the median OS of 19.0 months among treated metastatic patients in our cohort is longer than the 8 to 15 months reported historically and clearly reflects the benefit of both ICIs and MET TKIs.^6^^,^^21^

Treatment outcomes for TKIs in our cohort were much worse than expected, although crizotinib, rather than selective MET TKIs, was most used. Interestingly, whereas the ORR to crizotinib was comparable with prospective studies, the median PFS was much shorter in our cohort.^9^ A likely explanation is the high proportion of patients with poor ECOG performance status, reflective of a real-world population. There was also a high proportion of TKI-treated patients with sarcomatoid histology (18%) compared with other reports.^7^, ^8^, ^9^ Given the small patient numbers, we could not assess whether an association exists between histologic subtypes and TKI response. It was difficult to assess treatment outcomes for selective MET TKIs because many received a previous TKI. However, as no responses were seen with a second TKI, a switch in drug class should be considered among patients with progressive disease who remain fit for systemic therapy.

Patients who received PD-1 inhibitors exhibited favorable outcomes that are comparable to those of a wild-type population in the first-line setting.^11^^,^^12^^,^^15^ Concerns exist regarding poor ICI activity in oncogene-addicted NSCLC. Whereas it is quite clear that ICIs are inferior in *EGFR-* and *ALK-*mutant NSCLC, data for other oncogenic drivers are more controversial.^19^ The demographic patterns for *MET*ex14 skipping NSCLC are also different than for other driver mutations. Earlier retrospective studies reported low ORRs ranging from 7% to 17% with a median PFS of approximately 3 months.^18^^,^^19^^,^^21^ A potential explanation for these differences may be related to the line of treatment; in our cohort, 72% of patients received immunotherapy in the first-line setting compared with later-line settings in other retrospective studies.^18^^,^^19^^,^^21^ Furthermore, the reported ranges in response and PFS are what is expected with ICIs alone in the second-line setting in a wild-type population.^22^^,^^23^ Another very intriguing finding is the number of longer-term responders in our cohort, even among nonsmokers.

Current guidelines recommend either ICIs or MET-selective TKIs as first-line options for patients with *MET*ex14 skipping NSCLC. In our exploratory analysis of treatment sequencing, survival seemed to be improved when patients received ICIs first rather than a MET TKI. This analysis was limited by the use of crizotinib rather than the newer-generation MET TKIs. Nevertheless, it is an important finding because ICIs can be given empirically without the need to wait for NGS results, particularly in symptomatic patients. The tolerability and potential for durable responses with ICIs are attractive features for ICI use in the first-line setting. A prospective phase 2 study combining pembrolizumab and capmatinib exhibited increased rates of grade 3 and higher toxicities without any improvement in survival.^24^ Our study also revealed an increase in toxicity, particularly hepatitis, in patients receiving a MET TKI after PD-1 inhibitors. Careful monitoring is necessary as there was no clear association with the timing of the MET TKI. Severe toxicities were seen even after a washout period of 30 days or longer from ICIs.

Ongoing efforts in biomarker development may be helpful in guiding treatment sequencing. In our limited subset of patients, there was no correlation among the mutation type, such as acceptor or donor splice site, and response to TKIs. Other published studies, thus far, have not identified any correlations among mutation type, region, or copy number with sensitivity to TKIs.^25^ Interestingly, the MET protein (which, alone, is not predictive), when expressed in the presence of a *MET*ex14 skipping mutation, was associated with response to MET TKIs.^25^^,^^26^ To predict responses to ICIs, PD-L1 expression remains the main biomarker.^27^ In our series, high PD-L1 expression seems to be more prevalent than expected in a wild-type population with response rates similar to those expected for patients with PD-L1 greater than or equal to 50%. Other studies have explored tumor mutational burden in *MET*ex14 skipping-specific populations and did not find an association with response.^18^ To understand the mechanisms of response to different therapies, correlative analysis using archival tumor tissue of this patient series is underway.

Our case series is one of the largest cohorts with contemporary treatment outcomes data in NSCLC with *MET*ex14 skipping mutations. However, our findings must be interpreted within the limitations of this study. The overall sample size, especially when divided by treatment type, remains small. There may be inherent survivorship bias; reflexive NGS is not routinely performed. Our interpretation of selective MET TKI efficacy in the real world is also limited because capmatinib and tepotinib were only available to previously treated patients during the study period. However, our findings with respect to crizotinib are in line with previous studies. The analysis of treatment sequencing remains hypothesis-generating but provides reassuring data that empirical treatment with ICIs is effective.

In summary, our study found that NSCLC with *MET*ex14 skipping mutations has an aggressive behavior with early recurrences after resection and rapid progression. We advocate for comprehensive genomic profiling that includes RNA-based testing in all patients because 9% of alterations in our population were identified as fusion-only events. Future development of *MET*-specific fluorescence in situ hybridization probes may improve access to testing. Whereas optimal treatment sequencing and biomarker selection remain unanswered by our study, we found a clear benefit of both ICI and MET TKI therapy. Ensuring testing for *MET*ex14 skipping and an early start on effective systemic therapy are two of the most important clinical management strategies in improving patient survival.

## CRediT Authorship Contribution Statement

**Sally C. M. Lau:** Conceptualization, Methodology, Formal analysis, Investigation, Resources, Dara curation, Writing – original draft, Writing – review and editing, Visualization, Project administration.

**Kirstin Perdrizet:** Conceptualization. Methodology, Investigation, Resources, Dara curation, Writing – review and editing.

**Andrea S. Fung:** Methodology, Investigation, Resources, Dara curation, Writing – review and editing.

**Danilo Giffoni M. M. Mata:** Methodology, Investigation, Resources, Dara curation, Writing – review and editing.

**Jessica Weiss:** Software, Validation, Formal analysis, Writing – review and editing, Visualization.

**Nick Holzapfel:** Investigation, Resources, Dara curation, Writing – review and editing.

**Geoffrey Liu:** Writing – review and editing.

**Penelope A. Bradbury:** Writing – review and editing.

**Frances A. Shepherd:** Writing – review and editing.

**Adrian G. Sacher:** Writing – review and editing.

**Harriet Feilotter:** Writing – review and editing.

**Brandon Sheffield:** Conceptualization, Writing – review and editing.

**David Hwang:** Conceptualization, Writing – review and editing.

**Ming Sound Tsao:** Conceptualization, Writing – review and editing.

**Susanna Cheng:** Conceptualization, Writing – review and editing.

**Parneet Cheema:** Conceptualization, Writing – review and editing.

**Natasha B. Leighl:** Conceptualization, Writing – review and editing, Supervision, Funding acquisition.
