# Supplementary material for: Programmed Cell Death Protein 1 Inhibitors and MET Targeted Therapies in NSCLC With MET Exon 14 Skipping Mutations: Efficacy and Toxicity as Sequential Therapies
Source: JTO Clin Res Rep. 2023 Aug 19;4(10):100562. doi: 10.1016/j.jtocrr.2023.100562 (PMC10514105; doi:10.1016/j.jtocrr.2023.100562)
Supplement: Supplementary Tables [file mmc1.docx]

**Supplemental Table 1. Clinical characteristics of individual patients who received a MET TKI.**

|  | TKI-treated  n=28 |
| --- | --- |
| Median age, years | 72 (range 49–89) |
| Sex  Female  Male | 19 (68)  9 (32) |
| Ethnicity  Caucasian  Asian  Other/Unknown | 18 (64)  7 (25)  3 (11) |
| Smoking  Non-smoker  Smoker | 21 (75)  7 (25) |
| Histology  Adenocarcinoma  Squamous  Pleomorphic/Sarcomatoid carcinoma  Other | 21 (75)  1 (4)  5 (18)  1 (4) |
| Stage at Diagnosis  1  2  3  4 | 2 (7)  0  3 (11)  23 (82) |
| ECOG PS at Diagnosis  0  1  ≥2 | 3 (11)  19 (68)  6 (21) |
| *MET* exon 14 aberration  Acceptor splice site  Donor splice site  Fusion  Other  Not reported | 3 (11)  16 (57)  1 (4)  2 (7)  6 (21) |

ECOG PS, Eastern Cooperative Oncology Group performance status; TKI, tyrosine kinase inhibitor.

**Supplemental Table 2. Clinical characteristics of individual patients who received a PD-1 inhibitor.**

|  | ICI-treated  n=25 |
| --- | --- |
| Median age, years | 70 (54–82) |
| Sex  Female  Male | 16 (64)  9 (36) |
| Ethnicity  Caucasian  Asian  Other/Unknown | 17 (68)  3 (12)  5 (20) |
| Smoking  Non-smoker  Smoker | 14 (56)  11 (44) |
| Histology  Adenocarcinoma  Squamous  Pleomorphic/Sarcomatoid carcinoma  Other | 18 (72)  0  4 (16)  3 (12) |
| Stage at Diagnosis  1  2  3  4 | 1 (4)  0  5 (20)  19 (76) |
| ECOG PS at Diagnosis  0  1  ≥2 | 5 (20)  18 (72)  2 (8) |
| PD-L1 Expression  ≥50%  1-49%  <1% | 18 (72)  5 (20)  2 (8) |
| *MET* exon 14 aberration  Acceptor splice site  Donor splice site  Fusion  Unknown | 4 (16)  13 (52)  3 (12)  5 (20) |

ECOG PS, Eastern Cooperative Oncology Group performance status; ICI, immune checkpoint inhibitor; PD-1, programmed cell death protein 1; PD-L1, programmed cell death ligand-1.
